# Unique organization of photosystem II supercomplexes and megacomplexes in Norway spruce

**DOI:** 10.1111/tpj.14918

**Published:** 2020-08-01

**Authors:** Roman Kouřil, Lukáš Nosek, Monika Opatíková, Rameez Arshad, Dmitry A. Semchonok, Ivo Chamrád, René Lenobel, Egbert J. Boekema, Petr Ilík

**Affiliations:** ^1^ Department of Biophysics Centre of the Region Haná for Biotechnological and Agricultural Research Faculty of Science Palacký University Šlechtitelů 27 Olomouc 783 71 Czech Republic; ^2^ Electron Microscopy Group Groningen Biomolecular Sciences and Biotechnology Institute University of Groningen Nijenborgh 7 Groningen 9747 AG The Netherlands; ^3^ Department of Protein Biochemistry and Proteomics Centre of the Region Haná for Biotechnological and Agricultural Research Faculty of Science Palacký University Šlechtitelů 27 Olomouc 783 71 Czech Republic

**Keywords:** clear native polyacrylamide electrophoresis, *Picea abies*, *Pinus sylvestris*, photosystem II, megacomplex, supercomplex, single‐particle electron microscopy, grana membrane

## Abstract

Photosystem II (PSII) complexes are organized into large supercomplexes with variable amounts of light‐harvesting proteins (Lhcb). A typical PSII supercomplex in plants is formed by four trimers of Lhcb proteins (LHCII trimers), which are bound to the PSII core dimer via monomeric antenna proteins. However, the architecture of PSII supercomplexes in Norway spruce[*Picea abies* (L.) Karst.] is different, most likely due to a lack of two Lhcb proteins, Lhcb6 and Lhcb3. Interestingly, the spruce PSII supercomplex shares similar structural features with its counterpart in the green alga *Chlamydomonas reinhardtii* [Kouřil *et al*. (2016) *New Phytol*. **210**, 808–814]. Here we present a single‐particle electron microscopy study of isolated PSII supercomplexes from Norway spruce that revealed binding of a variable amount of LHCII trimers to the PSII core dimer at positions that have never been observed in any other plant species so far. The largest spruce PSII supercomplex, which was found to bind eight LHCII trimers, is even larger than the current largest known PSII supercomplex from *C. reinhardtii*. We have also shown that the spruce PSII supercomplexes can form various types of PSII megacomplexes, which were also identified in intact grana membranes. Some of these large PSII supercomplexes and megacomplexes were identified also in *Pinus sylvestris*, another representative of the Pinaceae family. The structural variability and complexity of LHCII organization in Pinaceae seems to be related to the absence of Lhcb6 and Lhcb3 in this family, and may be beneficial for the optimization of light‐harvesting under varying environmental conditions.

## Significance Statement

Norway spruce is known to lack two Lhcb proteins (Lhcb6 and Lhcb3), and its photosystems II (PSII) are organized into supercomplexes, which are similar to those observed in the green alga *Chlamydomonas reinhardtii*. Here we show that PSII in spruce can bind even more light‐harvesting complexes than PSII in Chlamydomonas, which makes it the largest PSII supercomplex ever observed, and that spruce supercomplexes can form various megacomplexes, detectable also in intact grana membranes.

## INTRODUCTION

Photosystem II (PSII) is a large multi‐subunit pigment−protein complex embedded in the thylakoid membrane of cyanobacteria, algae and plants. It is a key player in light reactions of photosynthesis due to its ability to split water into oxygen, protons and electrons, which are further utilized in photosynthetic reactions (Barber, 2003).

A core complex of PSII forms a dimer (C_2_), which contains pigments and redox cofactors necessary for the photochemical reactions. In land plants, C_2_ associates with light‐harvesting complex II (LHCII), consisting of a variable number of membrane‐embedded light‐harvesting proteins (Lhcb1−6). The variability of LHCII composition and size is important for the optimization of the absorption cross‐section of the PSII core complex under different light conditions (Bailey *et al*., 2001; Ballottari *et al*., 2007; Kouřil *et al*., 2013; Albanese *et al*., 2016). Lhcb1−3 proteins are present only in the trimeric form (Jansson, 1994). Lhcb1 and Lhcb2 can form homotrimers, but they are also able to form heterotrimers with each other or with Lhcb3. These trimers specifically bind to C_2_ core with the help of monomeric proteins Lhcb4−6 (also called CP29, CP26 and CP24, respectively).

Depending on the strength of the association of the trimers to C_2_, we distinguish between strongly (S), moderately (M) and loosely (L) bound LHCII trimers (Dekker and Boekema, 2005; Kouřil *et al*., 2012, 2018; see also Figure 5). The S trimer consists of Lhcb1 and Lhcb2 proteins at different ratios and is attached to C_2_ via Lhcb5 and Lhcb4 proteins. The M trimer is formed by one copy of Lhcb3 and two Lhcb1/2 proteins (Caffarri *et al*., 2004, 2009; Su *et al*., 2017; Crepin and Caffarri, 2018). Lhcb3 is a structurally important component of the M trimer (Caffarri *et al*., 2009; Su *et al*., 2017), as it can interact with Lhcb6, one of the minor antenna proteins. Lhcb6, together with Lhcb4, plays a crucial role in the binding of M trimer to C_2_ (Kovács *et al*., 2006; de Bianchi *et al*., 2008, 2011).

In plants grown under optimal light conditions, the most abundant form of PSII is the C_2_S_2_M_2_ supercomplex, containing two strongly and two moderately bound trimers (Kouřil *et al*., 2013). Namely in plants grown under high light conditions, the size of the supercomplex can be reduced to C_2_S_2_, the smallest physiologically relevant form of PSII that binds just two S trimers (Morosinotto *et al*., 2006; Ballottari *et al*., 2007; Kouřil *et al*., 2013; Albanese *et al*., 2016). On the other hand, the C_2_S_2_M_2_ supercomplex can be occasionally further extended by the presence of an L trimer; however, the C_2_S_2_M_2_L_1‐2_ supercomplexes are very rare. Up to today, they have been found only in spinach and only as a very minor fraction of all supercomplexes (Boekema *et al*., 1999a,b). Recently, an additional LHCII trimer was found to be associated with C_2_S_2_M_2_ in Arabidopsis as well, but in a different position than in spinach and only as a part of PSII megacomplexes (Nosek *et al*., 2017).

Our understanding of the assembly and structure of plant PSII supercomplexes has significantly increased during the last 10 years. High‐resolution structures uncovered details of subunit positions and arrangement of pigment molecules, which are crucial for the identification of possible energy transfer pathways within the PSII supercomplex (Caffarri *et al*., 2009; Wei *et al*., 2016; van Bezouwen *et al*., 2017; Su *et al*., 2017). At the same time, the generally accepted hypothesis that the architecture of the PSII supercomplex is uniform in land plants has been recently disproved by our work showing the surprising absence of Lhcb3 and Lhcb6 proteins in some gymnosperm genera (Kouřil *et al*., 2016). The lack of these proteins, which had been considered as essential components of LHCII in all land plants, has apparent consequences for the structure of PSII supercomplex in these species, including Norway spruce (*Picea abies*; Kouřil *et al*., 2016).

Structural analysis of PSII supercomplex from spruce provided direct evidence that the M trimer (or rather the pseudo‐M trimer without Lhcb3) can bind to C_2_ even in the absence of Lhcb6 (Kouřil *et al*., 2016). However, the absence of Lhcb3 and Lhcb6 changes the orientation of the M trimer with respect to the C_2_ core, and this unique position of the M trimer has never been observed in any other land plant species. Interestingly, the orientation of the M trimer in spruce is similar to the position of the M trimer in the PSII supercomplex from the green alga *Chlamydomonas reinhardtii* (Kouřil *et al*., 2016). Compared with spruce, however, the PSII supercomplexes from *C. reinhardtii* are larger, because they contain two additional LHCII trimers and form C_2_S_2_M_2_N_2_ supercomplexes (Tokutsu *et al*., 2012; Drop *et al*., 2014; Shen *et al*., 2019; Sheng *et al*., 2019). The two additional trimers attached to the C_2_S_2_M_2_ in the alga were designated as N (naked) trimers, because – unlike S and M trimers − they bind directly to C_2_ core without the involvement of any monomeric antenna (Drop *et al*., 2014). Actually, the N trimers bind to C_2_ at the position that is in land plants occupied by Lhcb6 (CP24; Drop *et al*., 2014). Therefore, as the absence of Lhcb6 seems to be a prerequisite of the binding of this additional trimer, it is reasonable to ask whether the N trimer can be found in the spruce PSII supercomplex as well.

Plant PSII supercomplexes exhibit variability not only in their composition and structure, but they can also form variable higher order structures in the thylakoid membrane. Neighboring individual supercomplexes can interact with each other, forming so‐called PSII megacomplexes, which can be isolated from thylakoid membranes using mild solubilization conditions. Different types of interactions then result in different long‐distance arrangement of supercomplexes within the thylakoids (Kirchhoff *et al*., 2004, 2008; Nosek *et al*., 2017). Random PSII organization, as well as highly ordered (crystalline) arrays of PSII, have been observed, each characterized by different interactions between the supercomplexes (for reviews, see Kouřil *et al*., 2012; Kirchhoff, 2013). Structural analysis revealed that pairs of neighboring PSII supercomplexes can interact in two ways. The first one involves interaction between stromal sides of PSII supercomplexes located in two adjacent grana membranes (Daum *et al*., 2010; Albanese *et al*., 2016, 2017; Su *et al*., 2017), the second one takes place in the membrane plane and is mediated by the interaction between LHCII and C_2_ (Nosek *et al*., 2017). The physiological relevance of the formation of PSII megacomplexes *in vivo* was supported by their identification on the level of the thylakoid membrane (Daum *et al*., 2010; Nosek *et al*., 2017).

In the present work, we study the consequences of the loss of Lhcb3 and Lhcb6 proteins for the organization of PSII supercomplexes and megacomplexes in Norway spruce. As the spruce C_2_S_2_M_2_ supercomplex shares some structural features with the PSII supercomplex from *C. reinhardtii*, we focused on the question whether the additional N trimers typical for this alga could attach also to spruce PSII supercomplex. As we have shown, the absence of the Lhcb6 and Lhcb3 proteins also results in a loss of a rectangular shape of the C_2_S_2_M_2_ supercomplex, which is typical for other land plants (Kouřil *et al*., 2016). We were thus interested in how the modified shape of spruce supercomplexes affects their ability to form megacomplexes and higher order assemblies in thylakoid membranes.

## RESULTS

### Separation of large spruce PSII assemblies

A necessary prerequisite of our search for larger assemblies of PSII complexes in Norway spruce was the optimization of the solubilization and separation protocols. The solubilization was achieved using detergent α‐DDM, which is milder than β‐DDM used in our previous study (Kouřil *et al*., 2016). The concentration of α‐DDM was optimized to maximize the yield of high‐molecular‐mass bands (PSII megacomplexes and larger PSII supercomplexes) in clear native−polyacrylamide gel electrophoresis (CN−PAGE). Interestingly, the optimal detergent: chlorophyll ratio (w/w) for spruce appeared to be 50, whereas similar optimization performed for separation of larger PSII assemblies from *Arabidopsis thaliana* led to a ratio of 20 (Nosek *et al*., 2017). Therefore, the solubilization protocol is not universal and should be optimized for each plant species separately.

**Figure 1:**
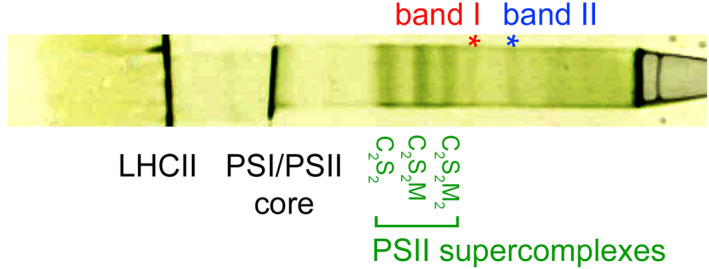
Separation of photosystem II (PSII) supercomplexes and megacomplexes from Norway spruce using clear native−polyacrylamide gel electrophoresis (CN−PAGE). Isolated thylakoid membranes were mildly solubilized by *n*‐dodecyl α‐d‐maltoside. The red and blue asterisks (the bands I and II) indicate the high‐molecular‐weight bands containing large PSII supercomplexes and megacomplexes, which were subjected to structural analysis by single‐particle electron microscopy. The bands of lower molecular weight represent different forms of PSII supercomplexes, PSI complex and PSII core complex, and LHCII proteins, respectively.

Figure 1 shows a typical separation profile of mildly solubilized thylakoid membranes from Norway spruce using CN−PAGE. Most PSII supercomplexes were separated into three dense bands in the central part of the gel. They differed in their antenna size and were assigned as the C_2_S_2_M_2_, C_2_S_2_M and C_2_S_2_ supercomplexes, in analogy with our previous paper (Kouřil *et al*., 2016). The use of mild detergent α‐DDM and optimized solubilization conditions allowed us to observe two additional high‐molecular‐weight bands in the upper part of the gel (the bands I and II; Figure 1). These bands contain larger PSII supercomplexes and megacomplexes, as mass spectrometry (MS) analysis revealed a high abundance of the proteins related to PSII and LHCII in these bands (Table S1). Both bands were excised from the gel, protein content was extracted by spontaneous elution and the obtained protein solution was subjected to structural analysis by single‐particle electron microscopy.

### Structural characterization of PSII supercomplexes

Image analysis of particle projections selected from electron micrographs revealed that PSII supercomplexes are present in both high‐molecular‐weight CN−PAGE bands (the bands I and II; Figure 1). Although various forms of large supercomplexes were found predominantly in band I, the largest PSII supercomplexes were so big that they co‐migrated with PSII megacomplexes in band II.

**Figure 2:**
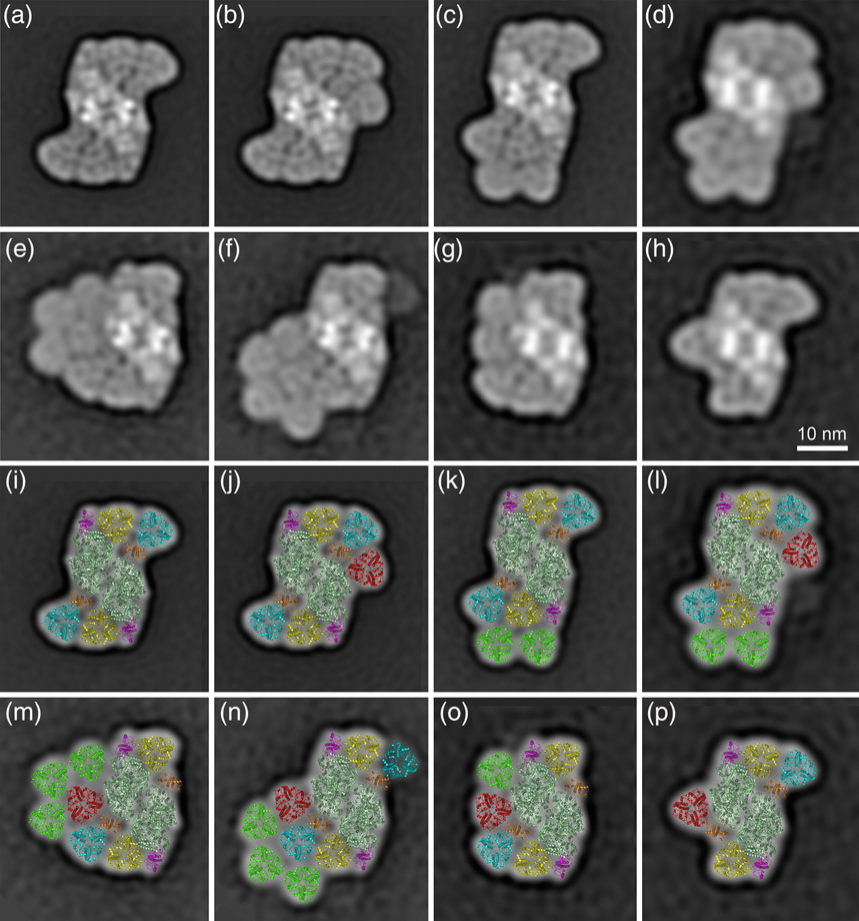
The large photosystem II (PSII) supercomplexes from Norway spruce. The supercomplexes were eluted from the band I (a–c, g, h) and the band II (d–f) in Figure 1. Projection maps of individual types of the PSII supercomplexes represent the best class averages of: (a) 12 015; (b) 9847; (c) 6356; (d) 622; (e) 1298; (f) 1018; (g) 1554; (h) 1219 particles. (i–p) Structural models of PSII supercomplexes were obtained by a fit of the high‐resolution structure (van Bezouwen *et al*., 2017). Individual PSII subunits are color‐coded: dark green – core complex; yellow – S trimer; cyan – M trimer; red – N trimer; green – L trimer; magenta – Lhcb5; orange – Lhcb4.

Single‐particle analysis of the samples prepared from band I resulted in the selection of 163 447 particle projections that were subsequently classified into 80 classes (Figure S1). In addition to the standard spruce C_2_S_2_M_2_ supercomplex, where the core complex C_2_ binds four LHCII trimers (Figure 2a), we observed novel types of PSII supercomplexes with up to six LHCII trimers (Figure 2b,c,g,h). The identified forms of PSII supercomplexes (Figure S1) differ significantly in their size, although they were obtained from the same highly focused band after CN−PAGE. Thus, it is probable that the smaller supercomplexes in Figure S1 (e.g. C_2_S_2_) are degradation products of the larger supercomplexes, which disintegrated during the elution of supercomplexes from the gel and/or during the preparation of samples for electron microscopy.

The samples obtained from band II (Figure 1) contained mainly PSII megacomplexes (Figure S2, detailed analysis follows), but a substantial part of the dataset contained various types of PSII supercomplexes. Except for several smaller PSII supercomplexes (C_2_S_2_, C_2_S_2_M and C_2_S_2_M_2_), which probably originate from the break‐up of less stable megacomplexes during the elution step and/or the specimen preparation, we observed very unique very large PSII supercomplexes that contain up to seven−eight LHCII trimers (Figure 2d–f), and have not been observed in any other plant species yet.

To examine the architecture of the large PSII supercomplexes and especially to analyze novel binding positions of LHCII trimers in detail, we fitted the electron microscopy projection maps with a recent molecular model of the PSII supercomplex (van Bezouwen *et al*., 2017). Structural models show the organization of LHCII trimers within the supercomplexes (Figure 2i–p). In addition to the standard S and M trimers (Figure 2i), in some supercomplexes we were able to identify the N trimers, typical for supercomplexes from *C. reinhardtii* (Figure 2j,l–p). The orientation of the N trimer with respect to C_2_ core was determined in the best‐resolved projection map of the C_2_S_2_M_2_N supercomplex (Figure 2b,j), and this orientation seems to be the same in all other supercomplexes but one (Figure 2l–o). The exception is the C_2_S_2_MN supercomplex (Figure 2p), where the orientation of the N trimer is different, probably due to the absence of the M trimer in the vicinity of the N trimer. Our analysis also revealed the presence of the L trimer in some of the supercomplexes (Figure 2m,o), and we were even able to see supercomplexes where there was a row of four LHCII trimers (S, M, N and L) around one side of the PSII core complex (Figure 2m,o). Moreover, our data show that the spruce PSII supercomplex has a unique ability to extend the antenna size even more by binding additional LHCII trimers. These trimers, which we term as the L_a_ trimers (additional loosely bound trimers), bind to the supercomplexes at different positions via the S, M, N and L trimers, and form the second row of LHCII trimers around the PSII core complex (Figure 2k–n).

To investigate whether the presence of larger PSII supercomplexes is a unique feature of Norway spruce or whether these structures can be found also in other members of the Pinaceae family (lacking the Lhcb3 and Lhcb6 proteins), we performed an analogical structural analysis of PSII supercomplexes isolated from Scots pine (*Pinus sylvestris*; Figure S3). Single‐particle image analysis revealed several larger forms of pine PSII supercomplexes (Figure S4a–f), which were identical to their counterparts in spruce (Figure 2a–d,h). This finding indicates that the unique ability to form larger antenna around the PSII core complex is likely a general property of the species from the Pinaceae family.

### Structural characterization of PSII megacomplexes

The inspection of electron micrographs of the sample prepared from the high‐molecular‐weight band depicted as band II (Figure 1) resulted in the selection of 66 650 particle projections, which were subjected to image analysis and classification into 64 classes (Figure S2). Visual analysis of the classification file (Figure S2) allowed us to estimate that the PSII megacomplexes account for about 30% of the whole dataset. Approximately half of the dataset contained various types of PSII supercomplexes (see above). The rest of the structures (about 20%) in Figure S2 contained other types of unspecific protein complexes (e.g. dimers/monomers of ATP synthase), which co‐migrated with the PSII megacomplexes in the gel.

**Figure 3:**
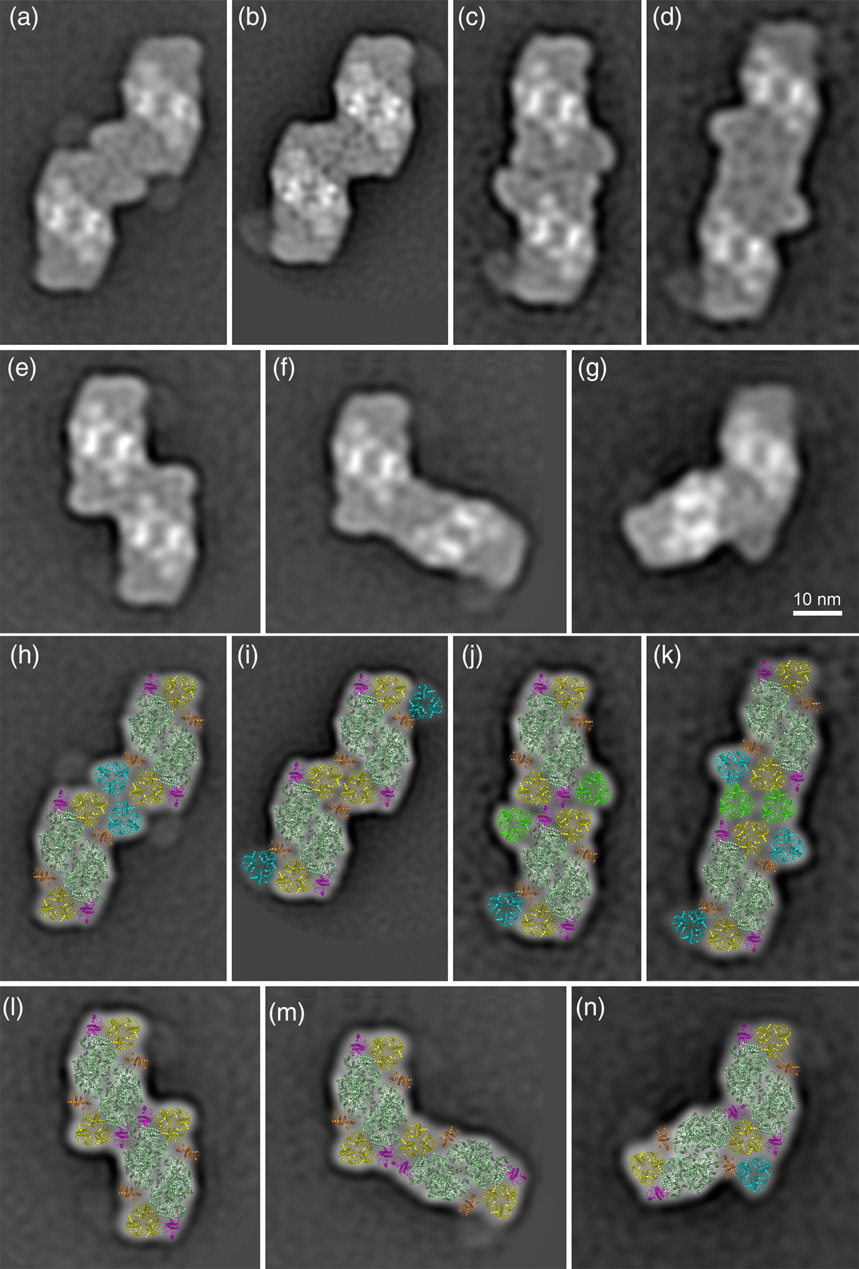
Various photosystem II (PSII) megacomplexes from Norway spruce. The megacomplexes were eluted from the band II (Figure 1). Projection maps of individual types of the PSII megacomplexes represent the best class averages of: (a) 1549; (b) 3118; (c) 1095; (d) 724; (e) 1178; (f) 915; (g) 1103 particles. (h–n) Structural models of the PSII megacomplexes obtained by a fit of the high‐resolution structure of PSII (van Bezouwen *et al*., 2017). Individual PSII subunits are color‐coded: dark green – core complex; yellow – S trimer; cyan – M trimer; green – L trimer; magenta – Lhcb5; orange – Lhcb4.

Figure 3 shows the best‐resolved classes of PSII megacomplexes. Each megacomplex consists of two copies of PSII supercomplexes, which associate with each other through their antenna complexes. Based on the orientation of PSII cores, the associations of the pairs of PSII supercomplexes were either parallel (Figure 3a–e) or non‐parallel (Figure 3f,g). The parallel associations were more abundant (70%) than the non‐parallel ones (30%).

Structural models of the projection maps of the PSII megacomplexes show that the megacomplexes are formed by variable interactions between C_2_S_2_M_2_ (Figure 3k), C_2_S_2_M (Figure 3h–k) and C_2_S_2_ (Figure 3j,l,m) supercomplexes. In some cases, PSII megacomplexes are formed with the help of the additional L trimers (Figure 3j,k). A closer view at the models of the identified megacomplexes (Figure 3) shows that the S trimer is most frequently involved in the megacomplex formation. Thus, the S trimer mediates probably the strongest binding between PSII supercomplexes within the PSII megacomplexes. However, also other subunits were found to participate in the association of supercomplexes into megacomplexes – Lhcb5 (CP26), Lhcb4 (CP29), C_2_, L and M trimers (in order of decreasing importance). Similar forms of PSII megacomplexes were also observed in Scots pine (Figure S4g–i).

### Interaction between PSII complexes in grana membranes

**Figure 4:**
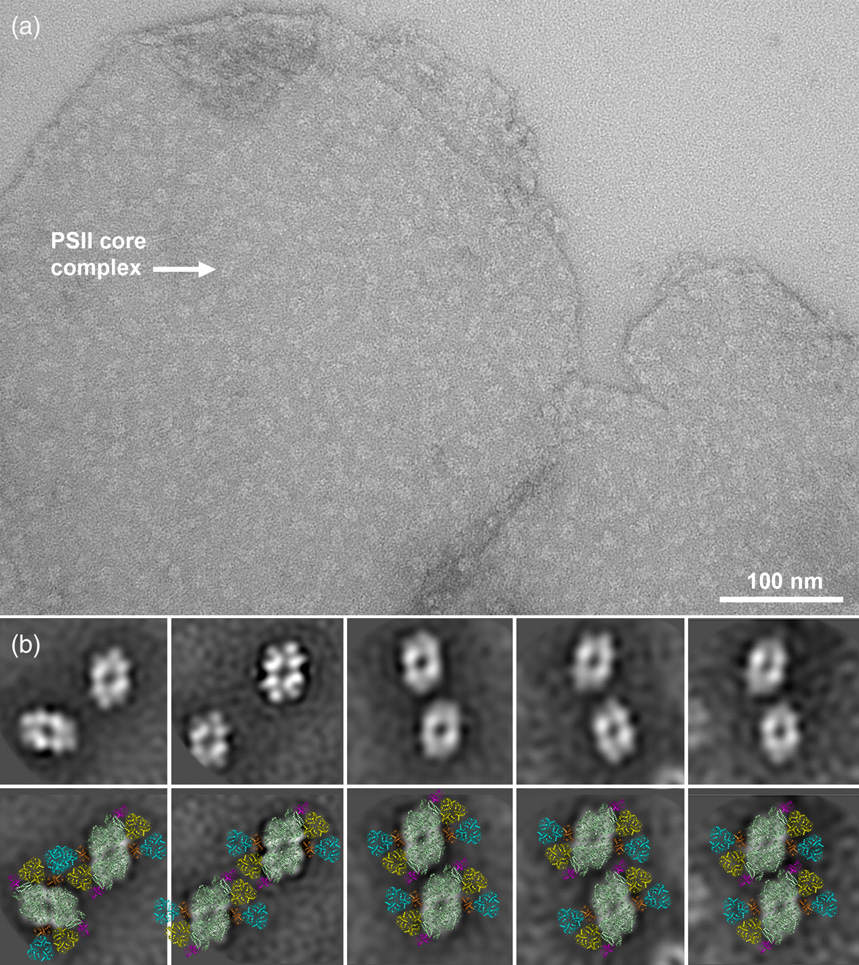
Distribution of photosystem II (PSII) complexes and their association into megacomplexes in isolated grana membranes. (a) An example of the electron micrograph of negatively stained grana membranes isolated from Norway spruce showing a density and distribution of PSII complexes. White arrow indicates a typical density of the PSII core complex. (b) Most abundant associations of PSII complexes into different types of megacomplexes found within the grana membranes after image analysis. (c) Structural models of the PSII megacomplexes obtained by a fit of the high‐resolution structure (van Bezouwen *et al*., 2017). Individual PSII subunits are color‐coded: dark green – core complex; yellow – S trimer; cyan – M trimer; magenta – Lhcb5; orange – Lhcb4.

Isolated grana membranes were analyzed using electron microscopy in order to characterize the organization and interaction between the neighboring PSII supercomplexes in the membrane. Visual screening of the electron micrographs revealed a random distribution of PSII complexes in the grana membranes (Figure 4a). The organization of PSII complexes into 2D crystalline arrays was not observed in analyzed micrographs, which is somewhat surprising as the array formation is quite common in other plant species (Kouřil *et al*., 2012). In order to confirm the physiological relevance of the PSII megacomplexes, which were observed after CN−PAGE separation, we investigated specific interactions between individual PSII complexes in the grana membranes. Indeed, image analysis of the PSII projections revealed several conserved mutual positions of PSII core complexes (Figure 4b). These pairs can be considered as PSII megacomplexes as the projection maps can be fitted with the model of the C_2_S_2_M_2_ supercomplex with tight interactions between the LHCII trimers or PSII core complexes (Figure 4c).

## DISCUSSION

### Structural characterization of large spruce PSII supercomplexes

Our previous study showed that the absence of Lhcb6 and Lhcb3 in the PSII complex of spruce results in a specific assembly of C_2_S_2_M_2_, which has never been found in land plants before (Kouřil *et al*., 2016). Here we show that the unique organization of the C_2_S_2_M_2_ supercomplex seems to be characteristic for the Pinaceae family, as we have found the identical architecture of this supercomplex also in Scots pine (Figure S4a). This unique organization of the PSII supercomplex brought several questions about the possible consequences for the attachment of additional LHCII trimers and the formation of larger super‐ and megacomplexes.

**Figure 5:**
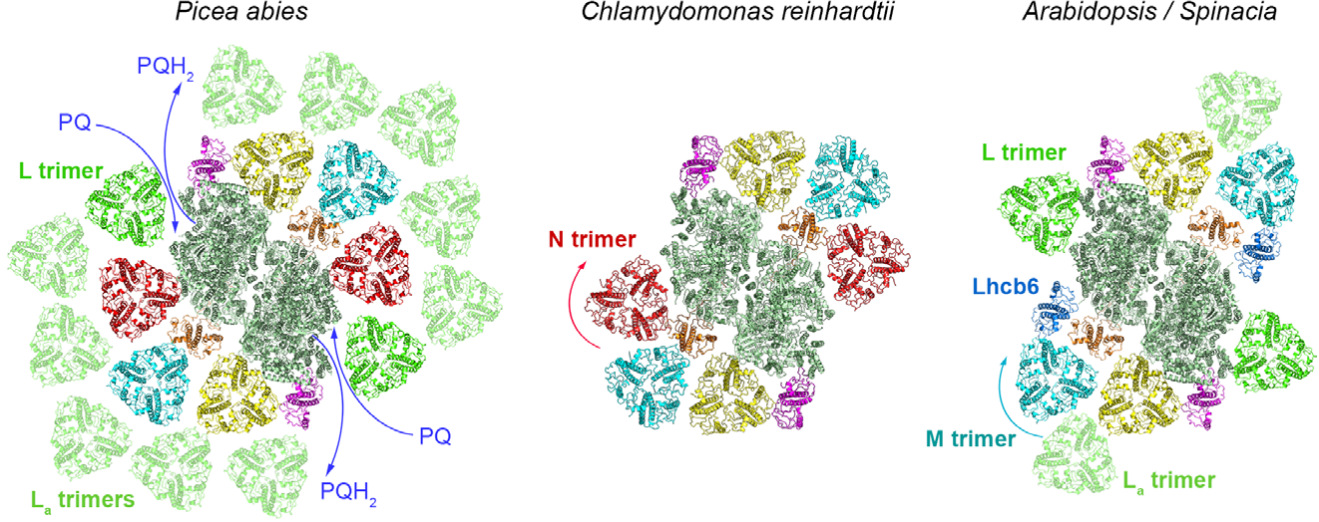
A hypothetical model of photosystem II (PSII) supercomplex from Norway spruce (*Picea abies*) and its comparison with evolutionary different organisms. The model of the complete PSII supercomplex in Norway spruce is based on the structures of different forms of PSII supercomplexes revealed by single‐particle electron microscopy. The specific orientation of the N trimer in Norway spruce probably enables a stable binding of the L trimer and formation of the second row of additional L_a_ trimers along the PSII core complex, but still keeps the path for plastoquinone molecules free. Different orientation of the N trimer in *Chlamydomonas reinhardtii* (Shen *et al*., 2019) likely does not support the binding of the L trimer. In the majority of land plants, the binding site for the N trimer is occupied by the Lhcb6 protein, which probably modifies the binding of the M trimer. The L trimer can very occasionally bind to the PSII core complex (e.g. in spinach; Boekema *et al*., 1999a,b) or additional L_a_ trimers can associate with PSII at the site of the S/M trimers (e.g. Arabidopsis; Nosek *et al*., 2017). Individual PSII subunits are color‐coded: dark green – core complex; yellow – S trimer; cyan – M trimer; red – N trimer; green – L trimer; light green – L_a_ trimers; magenta – Lhcb5; orange – Lhcb4.

As the spruce PSII supercomplex shares some features with PSII supercomplex in *C. reinhardtii* (Kouřil *et al*., 2016), the first hypothesis we wanted to verify was whether the spruce C_2_S_2_M_2_ supercomplex is able to bind additional LHCII trimer (N trimer) in the same way as *C. reinhardtii* (Tokutsu *et al*., 2012; Drop *et al*., 2014; Shen *et al*., 2019; Sheng *et al*., 2019). The outcome of single‐particle analysis unequivocally confirmed that the C_2_S_2_M_2_ supercomplex is indeed able to bind the N trimer; however, its precise orientation depends on the presence of the M trimer. In supercomplexes where both the N and M trimers are present (Figure 2j,l–o), the N trimer is rotated clockwise by 63° compared with *C. reinhardtii*. This rotation could be most probably explained by the differences in the structure of the M and N trimers themselves. While the LHCII trimers in Norway spruce are formed by Lhcb1 and Lhcb2 proteins (Kouřil *et al*., 2016) with possible involvement of Lhcb5 (Grebe *et al*., 2019), the LHCII trimers in *C. reinhardtii* are formed by Lhcbm1/2/3/6/7 proteins (Drop *et al*., 2014; Shen *et al*., 2019). In Scots pine, the orientation of the N trimer in PSII supercomplexes is the same as in spruce (Figure S4b,e). Interestingly, in both spruce and pine, we have also found supercomplex where the N trimer is bound to the PSII core without the presence of the M trimer (Figures 2p and S4f, respectively). In this case, the N trimer binds to the PSII core complex in the same orientation as in *C. reinhardtii* (Figure 5). This finding indicates that the mutual interaction between the M and N trimer determines the orientation of the N trimer in PSII supercomplexes in Pinaceae. However, due to the lack of high‐resolution structural details of the spruce and pine PSII supercomplexes, we cannot exclude the possibility that some PSII core subunits are involved in the binding of the N trimer – namely PsbH and PsbX, which are involved in the binding of the N trimer in *C. reinhardtii* (Shen *et al*., 2019; Sheng *et al*., 2019). A high‐resolution cryo‐electron microscopy structure of spruce/pine supercomplex will be necessary to unequivocally resolve this question.

The optimization of the separation procedure allowed us not only to find supercomplexes with attached N trimer, but also PSII supercomplexes that are even larger than the C_2_S_2_M_2_N. In some of these supercomplexes from Norway spruce, another LHCII trimer binds in the vicinity of the N trimer (Figure 2m,o). As the position of this trimer is similar to the position of the L trimer observed in the very small fraction of isolated PSII supercomplexes from spinach (Boekema *et al*., 1999a,b; see also Figure 5), we tentatively named it as the L trimer. Such a large PSII supercomplex with four LHCII trimers (S, M, N, L) bound around the PSII core has never been found in any plant species so far. There are some indications that this structure might exist in *C. reinhardtii*, as Kawakami *et al*. (2019) recently found that there are four types of LHCII trimers in *C. reinhardtii*. Nevertheless, their work was based on biochemical methods, and the attachment of the L trimer to the C_2_S_2_M_2_N_1‐2_ supercomplex in *C. reinhardtii* is yet to be confirmed experimentally. It is possible that the difference in the orientation of the N trimer in spruce and in *C. reinhardtii* affects the strength of the binding of the L trimer, making it more labile and thus more prone to dissociation in the latter.

Surprisingly, we have found out that even the large PSII supercomplex with attached S, M, N and L trimers is not the largest one that could be found in spruce. Our structural analysis revealed for the first time that the PSII supercomplex is able to bind LHCII trimers in two rows. The LHCII trimers of the second (outer) row, which we named L_a_ (additional L) trimers, can bind to PSII at five specific positions along the inner row of the S, M, N and L trimers. The largest spruce PSII supercomplex identified in our study binds seven or eight LHCII trimers (Figure 2l–n), which exceeds the antenna size of C_2_S_2_M_2_N_2_ supercomplex in *C. reinhardtii*, currently considered to be the largest known PSII supercomplex. Considering the twofold symmetry of the PSII supercomplex, we can hypothesize that the most complete spruce PSII supercomplex would have the ability to bind up to 18 LHCII trimers (Figure 5). The hypothetical model can be applied also for Scots pine, where the largest identified form of the PSII supercomplex binds seven LHCII trimers (Figure S4e). Interestingly, the antenna organization in this large PSII supercomplex would still keep free the path for plastoquinone to the acceptor sites in the PSII core complex (van Eerden *et al*., 2017), which gives the hypothetical model a physiological relevance.

### Structural characterization of spruce PSII megacomplexes

It is well known that plant PSII supercomplexes can further associate into larger assemblies, forming PSII megacomplexes. Our work shows that the species from Pinaceae family are no exception. Single‐particle electron microscopy analysis revealed several distinct types of spruce and pine PSII megacomplexes, which were formed either by parallel or non‐parallel associations between two PSII supercomplexes (Figures 3 and S4g–i). The main distinctive feature of the spruce and pine PSII megacomplexes, when compared with those from Arabidopsis (Nosek *et al*., 2017), is their lower structural variability. While we found only seven types of megacomplexes in spruce and three in pine, their number in Arabidopsis was significantly higher (13; Nosek *et al*., 2017). This finding can be simply explained by the lower stability of PSII megacomplexes in Pinaceae. This explanation is supported, for example, by our finding that during the image analysis of the sample from the high‐molecular‐weight band II (Figure 1), we have observed classes of the C_2_S_2_, C_2_S_2_M and C_2_S_2_M_2_ PSII supercomplexes, which are the building blocks of the megacomplexes (Figure S2). As these supercomplexes cannot co‐migrate in the gel with megacomplexes, which have roughly twice the size and weight, the only feasible explanation of the presence of these supercomplexes in this sample is that they originate from the break‐up of megacomplexes during the elution step and specimen preparation for electron microscopy.

In our previous study with Arabidopsis, we have successfully identified some forms of megacomplexes also on the level of the thylakoid membrane (Nosek *et al*., 2017), and therefore we have employed this approach also for spruce. Image analysis of the PSII distribution in thylakoid membranes from spruce revealed five well‐resolved specific associations between the adjacent PSII supercomplexes (Figure 4b,c), which strongly indicates that PSII megacomplexes in spruce exist *in vivo*. The proposed structural models of these megacomplexes show that they can be formed, for example, by C_2_S_2_M_2_ supercomplexes (Figure 4c), but we cannot exclude the involvement of even larger forms of the PSII supercomplexes. Three types of the megacomplexes represent an almost parallel association of the PSII supercomplexes along the core complex, which is similar to the megacomplex formations observed in Arabidopsis (Nosek *et al*., 2017).

It is, however, important to note that ‐ unlike in Arabidopsis (Nosek *et al*., 2017) – in spruce the PSII associations observed in thylakoid membranes do not correspond to any spruce PSII megacomplexes that we were able to isolate and separate. The interactions between the neighboring PSII supercomplexes in the membrane seem to be too weak to keep the megacomplexes intact during the separation procedure and/or specimen preparation for electron microscopy analysis. Thus, it is likely that the separated PSII megacomplexes (Figure 3) represent the most stable megacomplex forms, but they do not represent the most frequent type of megacomplexes found in the thylakoid membrane.

We assume that the lower stability of the bigger PSII megacomplexes could be closely related to the overall architecture of individual PSII supercomplexes. While a majority of the isolated spruce and pine megacomplexes are formed by pairs of smaller forms of PSII supercomplexes (C_2_S_2_ or C_2_S_2_M; Figures 3 and S4g–i), in Arabidopsis the megacomplexes are formed by the larger, C_2_S_2_M_2_, supercomplexes. Considering a different shape of the C_2_S_2_M_2_ supercomplexes in spruce and Arabidopsis (Figure 5), it becomes obvious that the rectangular shape of the C_2_S_2_M_2_ in Arabidopsis can provide more stable interaction between adjacent supercomplexes. Indeed, a majority of the Arabidopsis megacomplexes were formed by parallel associations of two PSII supercomplexes along their longer sides, which probably represents a relatively stable configuration. The shape of the C_2_S_2_M_2_ supercomplex in Pinaceae, modified by the absence of Lhcb3 and Lhcb6 proteins (Kouřil *et al*., 2016), cannot provide the same interaction interface, which could potentially explain the lower stability of the PSII megacomplexes.

In summary, the structural analysis of PSII from two species from the Pinaceae family, Norway spruce and Scots pine, further extended our knowledge about the architecture of PSII supercomplexes. Our results clearly show that there are more binding sites for LHCII trimers than it was originally thought. We suggest that the evolutionary loss of Lhcb6 in Pinaceae resulted in the ability of PSII core to bind the N trimer as in the case of green alga *C. reinhardtii* (Figure 5). A unique orientation of the N trimer in Pinaceae probably supports the binding of the L trimer and additional L_a_ trimers, thus leading to the formation of larger PSII supercomplexes. A different orientation of the N trimer in *C. reinhardtii* or the absence of the N trimer in other land plant species (e.g. Arabidopsis, spinach) likely results in a loose binding of the L trimer to the PSII core (Figure 5).

The interesting question to answer is what environmental conditions would lead to a formation of such giant PSII supercomplexes, hypothetically up to the C_2_S_2_M_2_N_2_L_2_L_a10_ supercomplex. Formation of these giant supercomplexes would require LHCII trimers to be in great excess of PSII core complexes. Acclimation of land plants to low light intensity is well known to induce a higher LHCII/PSII core ratio (Bailey *et al*., 2001; Ballottari *et al*., 2007; Kouřil *et al*., 2013); however, for example Norway spruce does not follow this strategy, as the antenna size remains almost unchanged during acclimation (Kurasová *et al*., 2003; Štroch *et al*., 2008). Nevertheless, there are indications that the acclimation of conifers to winter conditions can lead to the enhanced LHCII/PSII core ratio (Verhoeven, 2014), which may create favorable conditions for the formation of large PSII supercomplexes.

## EXPERIMENTAL PROCEDURES

### Plant material and isolation of PSII megacomplexes and supercomplexes

Norway spruce [*Picea abies* (L.) Karst.] and Scots pine [*Pinus sylvestris* (L.); Semenoles, Liptovský Hrádok, Slovakia] seedlings were grown in a growth chamber with 16 h: 8 h, light: dark photoperiod at 21°C for 4 weeks. Plants were illuminated with white light at 100 μmol photons m^−2^ sec^−1^ (400–700 nm). Isolation of thylakoid membranes and electrophoretic separation of solubilized membranes using CN−PAGE were performed according to a protocol described by Nosek *et al*. (2017) with one modification in the solubilization condition, that is spruce and pine thylakoid membranes with 10 μg of chlorophylls were solubilized with *n*‐dodecyl α‐d‐maltoside (α‐DDM) using a detergent: chlorophyll mass ratio of 50.

Spruce PSII membranes were isolated using mild solubilization of thylakoid membranes with digitonin (0.5 mg of chlorophylls per ml and 0.5% digitonin) in a buffer containing 20 mm HEPES, pH 7.5, 5 mm MgCl_2_ and 15 mm NaCl. Solubilization was performed for 20 min at 4°C with slow stirring, and was followed by centrifugation (5 min, 12 000 ***g***, 4°C). The pellet with the non‐solubilized PSII grana membranes was washed twice with the buffer mentioned above, spun down again for 5 min (12 000 ***g***, 4°C) and then used for electron microscopy analysis.

### Single‐particle electron microscopy and image analysis

The PSII megacomplexes and supercomplexes were eluted from excised CN−PAGE gel bands according to Kouřil *et al*. (2014). Obtained protein solutions were directly used for electron microscopy specimen preparations by negative staining with 2% uranyl acetate on glow‐discharged carbon‐coated copper grids. Electron microscopy of spruce PSII supercomplexes and megacomplexes was performed on a Tecnai G2 F20 microscope (FEI) equipped with a LaB_6_ cathode, operated at 200 kV. Images were recorded with an UltraScan 4000 UHS CCD camera (Gatan, Pleasanton, CA, USA) at 130 000 × magnification with a pixel size of 0.224 nm at the specimen level after binning the images to 2048 × 2048 pixels. GRACE software (Oostergetel *et al*., 1998) was used for semi‐automated acquisition of 14 000 and 11 000 images of PSII complexes eluted from the bands I and II, respectively, and two datasets of approx. 163 447 (band I) and 66 650 (band II) single‐particle projections were selected and subjected to several rounds of single‐particle image analysis and classification (Boekema *et al*., 2009) using RELION software (Scheres, 2012) and reference‐free 2D classification using SCIPION image processing framework (De La Rosa‐Trevín *et al*., 2016). Pseudo‐atomic models of obtained PSII projection maps were created using PYMOL (DeLano, 2002).

Electron microscopy of isolated grana membranes from Norway spruce and eluted PSII megacomplexes and supercomplexes from Scots pine was performed on a Tecnai G2 F20 microscope (FEI) equipped with a field emission gun operated at 200 kV. Images were recorded with an Eagle 4K CCD camera (FEI) at 83 000 × magnification with a pixel size of 0.36 nm (spruce grana membrane), and at 130 000 × magnification with a pixel size of 0.226 nm (pine PSII super‐/megacomplexes) at the specimen level after binning the images to 2048 × 2048 pixels. In total, 150 micrographs were recorded and about 13 000 manually selected projections of PSII particles were analyzed using RELION software (Scheres, 2012) to reveal the specific formation of PSII megacomplexes in the spruce grana membrane. Two datasets of approx. 73 000 and 310 000 single‐particle projections were selected from 8000 and 7000 electron micrographs of pine PSII megacomplexes and PSII supercomplexes, respectively, and analyzed using reference‐free 2D classification using SCIPION image processing framework (de la Rosa‐Trevín *et al*., 2016).

### Proteomic characterization of spruce PSII supercomplexes and megacomplexes

The electroeluted PSII super‐ and megacomplexes were first concentrated and transferred to a denaturing buffer using a centrifugal filter unit with 3K cut‐off. Next, in‐solution protein digestion was performed with commercially available trypsin, as described previously (León *et al*., 2013). The tryptic peptides were desalted and fractionated with the use of a custom reversed‐phase (C18) microcolumn (Franc *et al*., 2012) and subsequently analyzed by LC‐MS (Simerský *et al*., 2017). The acquired MS data were searched against *P. abies*‐specific protein database (Grebe *et al*., 2019) employing MaxQuant software v.1.6.10.43 (Beck *et al*., 2015; Tyanova *et al*., 2016) with Andromeda search engine (Cox *et al*., 2011). To evaluate the abundances of the identified proteins, the well‐established iBAQ method (Schwanhäusser *et al*., 2011) was applied. Missing protein annotations were assigned by pBLAST homology searches. Details for all described methods can be found in the Supporting Information (Methods S1).

## Author contributions

RK, LN, PI planned and designed the research. RK, LN, MO, RA, DS, IC, RL performed experiments. RK, LN, MO, RA, DS, IC, R.L., EJB and PI analyzed the data. RK and PI wrote the manuscript, and all authors revised and approved it.

## Conflict of interest

The authors have no conflict of interest to declare.

## Supporting information


**Figure S1.** Single‐particle image analysis and classification of PSII supercomplexes from Norway spruce extracted from CN−PAGE band I.

**Figure S2.** Single‐particle image analysis and classification of PSII supercomplexes and megacomplexes from Norway spruce extracted from CN−PAGE band II.

**Figure S3.** Separation of pigment−protein complexes from Scots pine using CN−PAGE.

**Figure S4.** Structural characterization of PSII supercomplexes and megacomplexes from Scots pine.

**Data S1**. Complete protein identification data and other files containing information related to protein identification as exported from MaxQuant software v. 1.6.10.43.

**Table S1.**An overview of protein composition of the PSII supercomplex and megacomplex bands (CN−PAGE bands I and II).

**Table S2.**A list of all identified proteins from CN−PAGE bands I and II with corresponding characteristics and annotations.

**Methods S1**. Proteomic characterization of Norway spruce PSII supercomplexes and megacomplexes.

## Data Availability

All relevant data can be found within the manuscript and its supporting materials. The mass spectrometry proteomics data have been deposited to the ProteomeXchange Consortium via the PRIDE (Perez‐Riverol *et al*., 2019) partner repository with the dataset identifier PXD020138.
