# Supplementary figures and images for: Unique organization of photosystem II supercomplexes and megacomplexes in Norway spruce
Source: Plant J. 2020 Aug 1;104(1):215–25. doi: 10.1111/tpj.14918 (PMC7590091; doi:10.1111/tpj.14918)

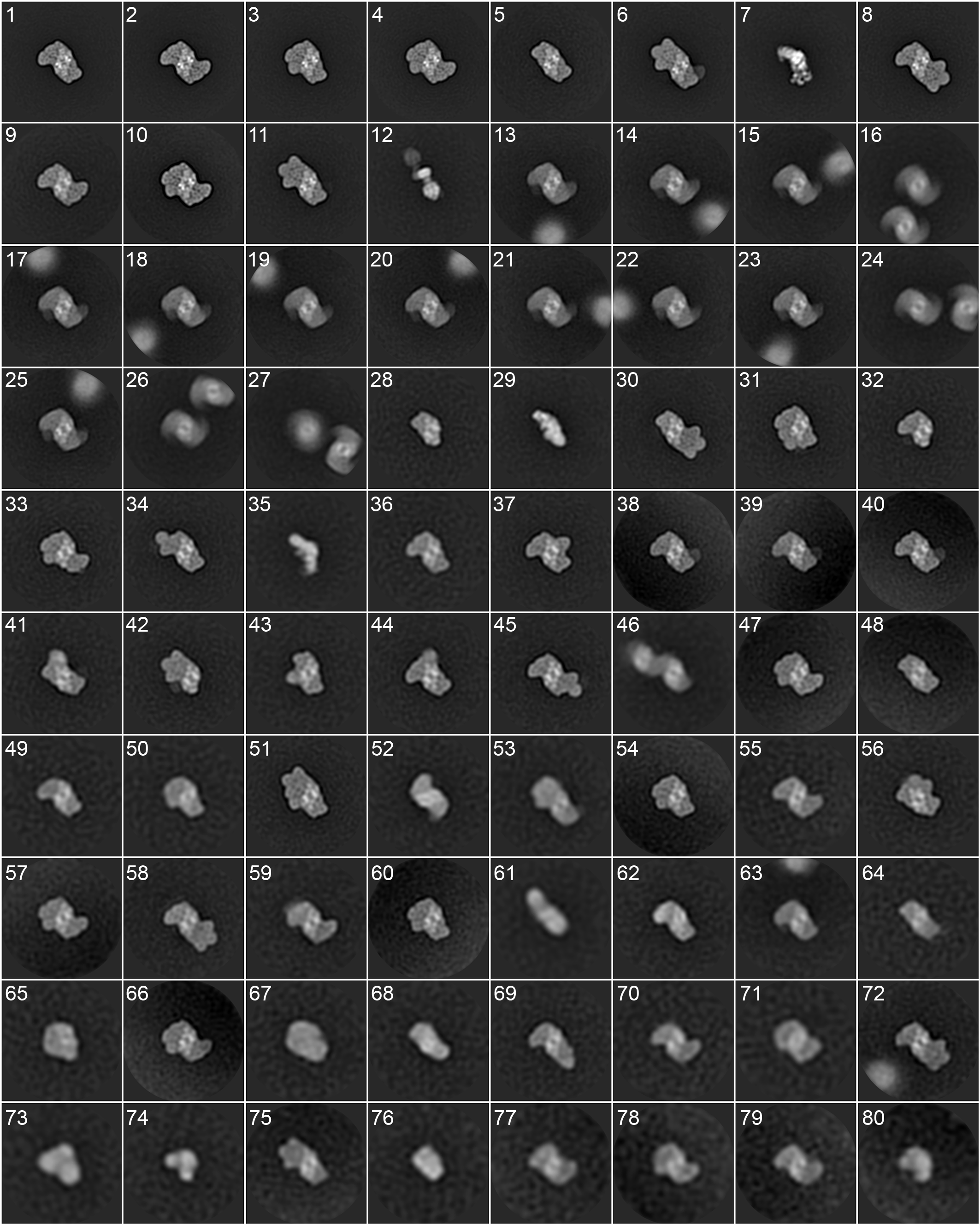

Supplement: Supplementary file 1 — Figure S1. Single‐particle image analysis and classification of PSII supercomplexes from Norway spruce extracted from CN−PAGE band I. [file TPJ-104-215-s001.tif]

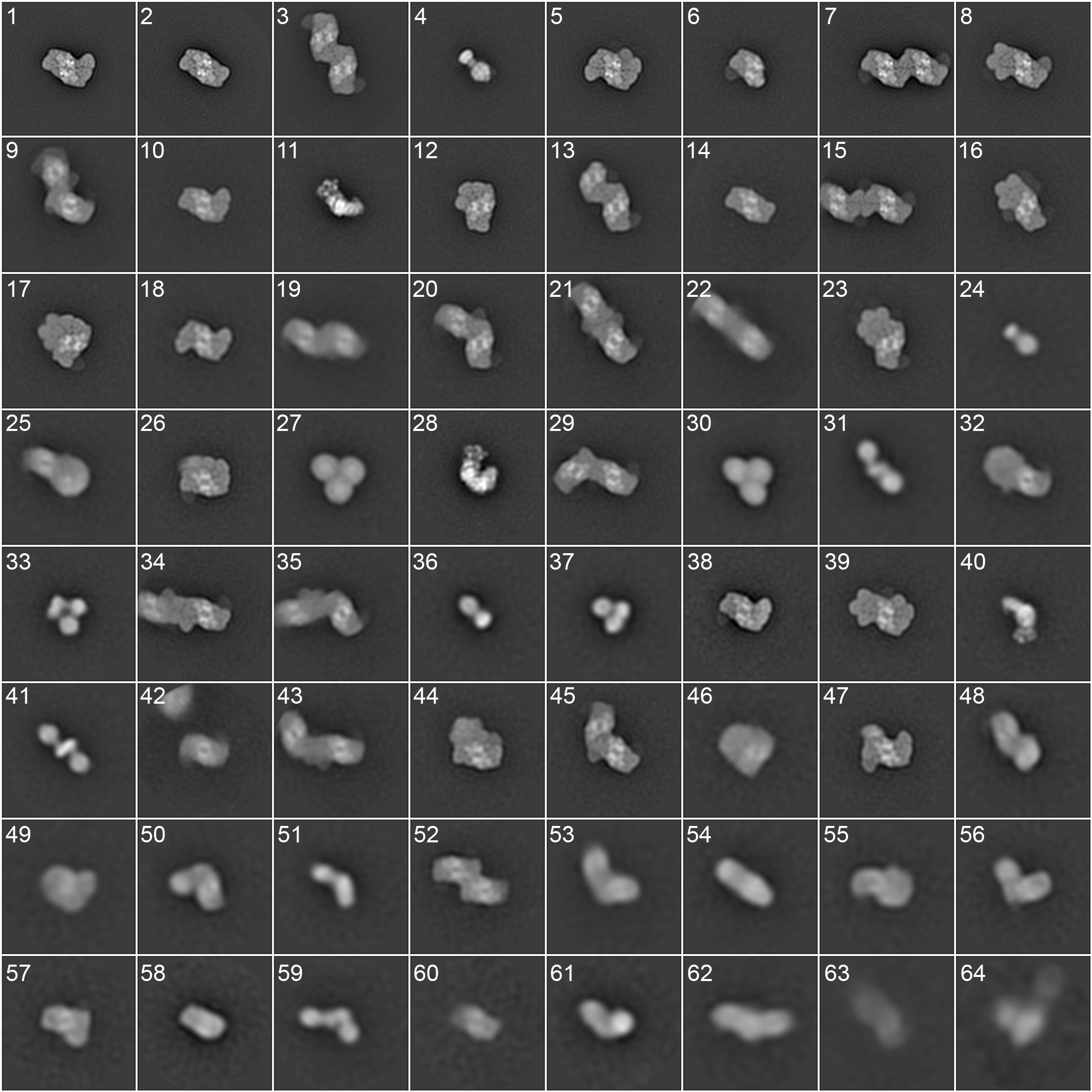

Supplement: Supplementary file 2 — Figure S2. Single‐particle image analysis and classification of PSII supercomplexes and megacomplexes from Norway spruce extracted from CN−PAGE band II. [file TPJ-104-215-s002.tif]

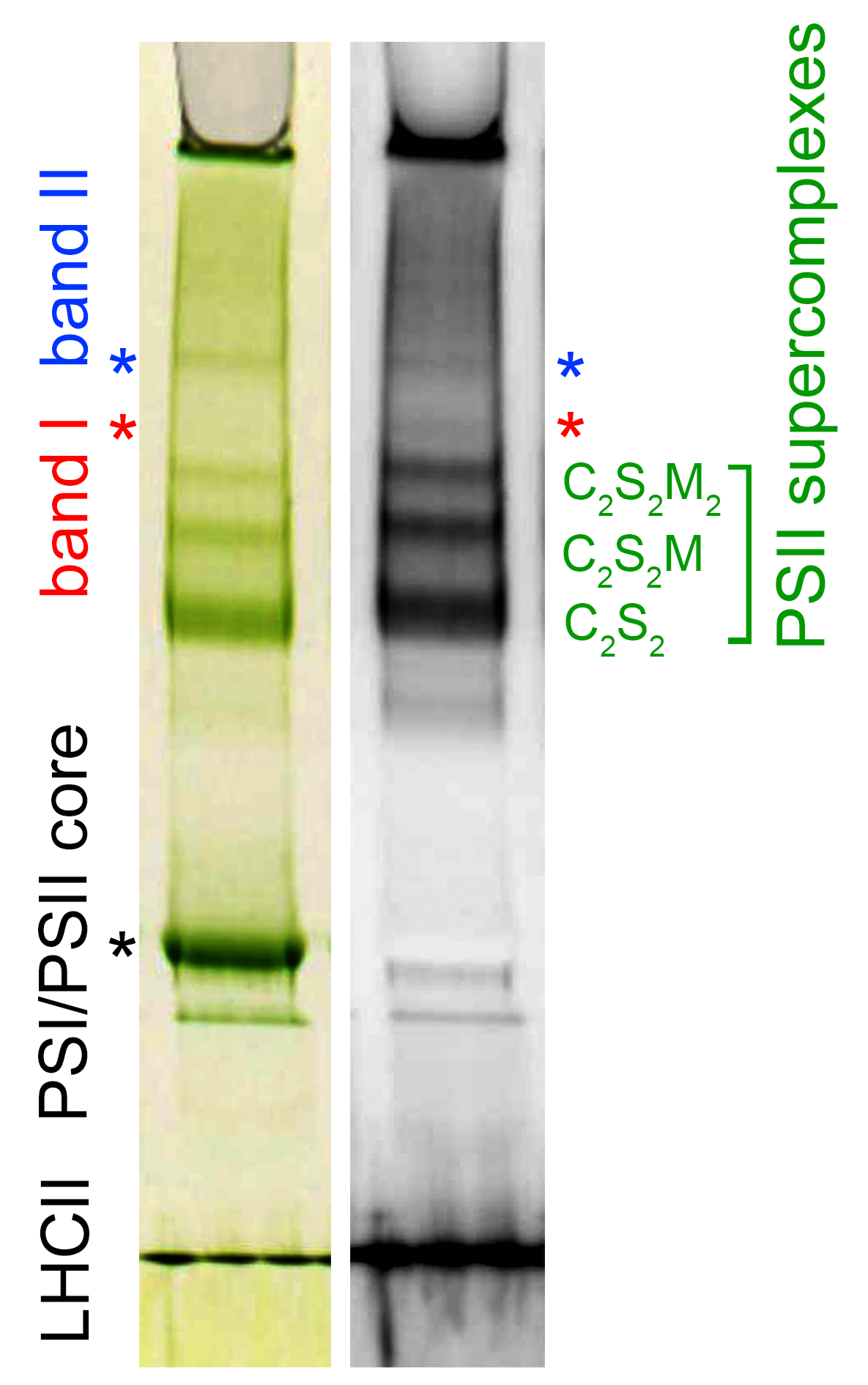

Supplement: Supplementary file 3 — Figure S3. Separation of pigment−protein complexes from Scots pine using CN−PAGE. [file TPJ-104-215-s003.tif]

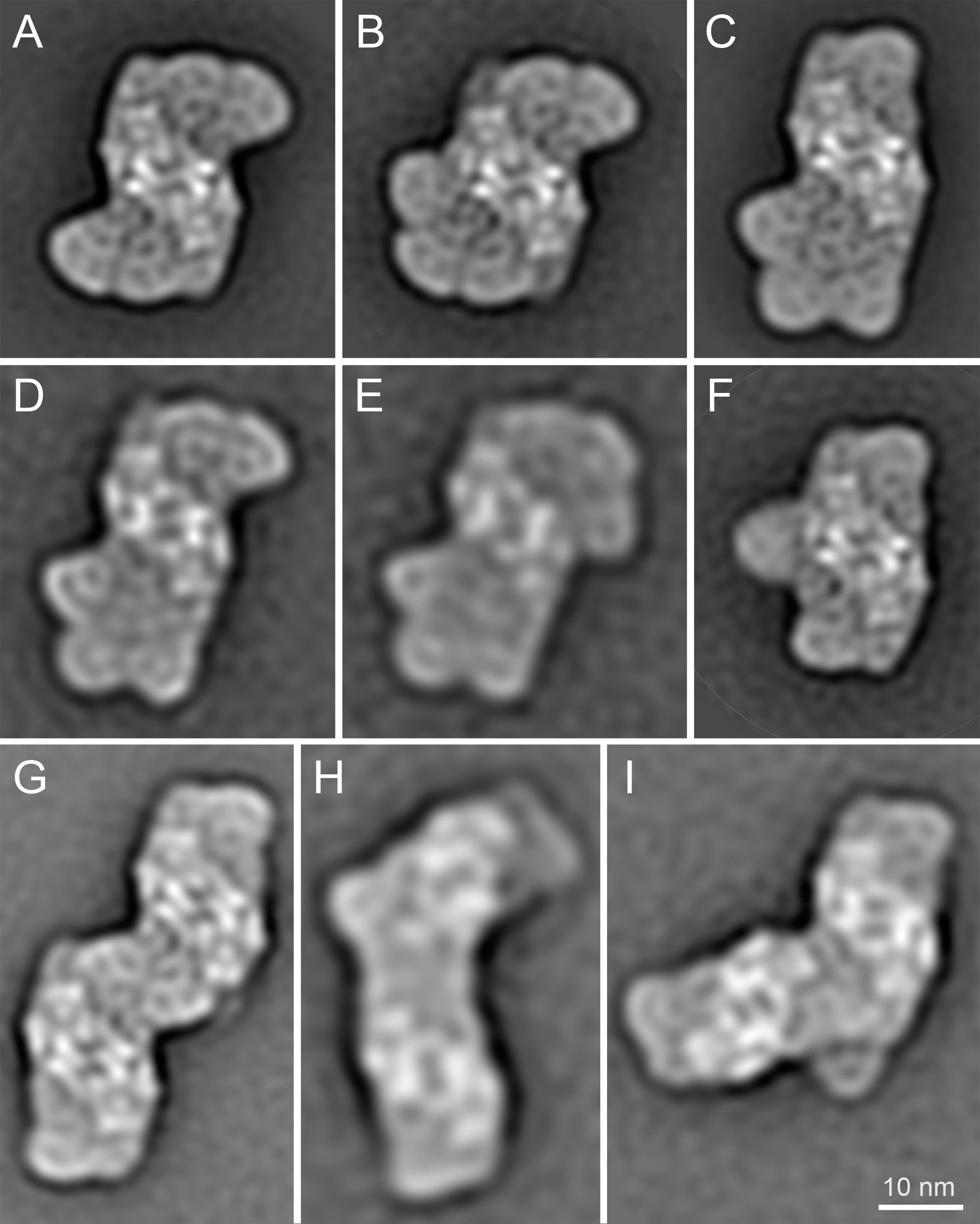

Supplement: Supplementary file 4 — Figure S4. Structural characterization of PSII supercomplexes and megacomplexes from Scots pine. [file TPJ-104-215-s004.tif]
