# Supplementary material for: Unique organization of photosystem II supercomplexes and megacomplexes in Norway spruce
Source: Plant J. 2020 Aug 1;104(1):215–25. doi: 10.1111/tpj.14918 (PMC7590091; doi:10.1111/tpj.14918)
Supplement: Supplementary file 6 — Table S1.An overview of protein composition of the PSII supercomplex and megacomplex bands (CN−PAGE bands I and II). [file TPJ-104-215-s006.pdf]

Supporting Table 1. An overview of protein composition of the PSII supercomplex and megacomplex bands (CN-PAGE bands I and II).

Protein category	Protein details	band I protein IDs[a]	band II protein IDs[a]	band I iBAQ[b] intensity	band II iBAQ[b] intensity	band I composition (%)	band II composition (%)
Chloroplast	PSII related	25	25	13420878	9634223	81.35	68.13
	Other	57	45	2802967	3669596	16.99	25.95
Plant contaminant	Mitochondrion	18	12	145159	81427	0.88	0.58
	Cytoplasm	6	4	73982	22095	0.45	0.16
	Nucleus	1	-	2592	-	0.02	-
	GA[c]	-	1	-	726600	-	5.13
	ER[d]	1	-	8354	-	0.05	-
	Apoplast	1	-	38657	-	0.23	-
	Peroxisome	1	-	n.a.	-	n.a.	-
	Storage protein	2	1	4571	7227	0.03	0.05
In total		112	88	16497160	14141168	100	100

[a]IDs – identifications; [b]iBAQ – intensity based absolute quantification; [c]GA – Golgi apparatus; [d]ER – endoplasmic reticulum
