# Supplementary material for: Unique organization of photosystem II supercomplexes and megacomplexes in Norway spruce
Source: Plant J. 2020 Aug 1;104(1):215–25. doi: 10.1111/tpj.14918 (PMC7590091; doi:10.1111/tpj.14918)
Supplement: Supplementary file 8 — Methods S1. Proteomic characterization of Norway spruce PSII supercomplexes and megacomplexes. [file TPJ-104-215-s008.docx]

**Supporting information**

**Supporting information Fig. S1** – Single-particle image analysis and classification of photosystem II supercomplexes from Norway spruce extracted from CN-PAGE band I.

Image analysis of 163447 particle projections, selected from electron micrographs of the band containing photosystem II supercomplexes, resulted in classification into 80 classes (Fig. S1).

**Supporting information Fig. S2** – Single-particle image analysis and classification of photosystem II supercomplexes and megacomplexes from Norway spruce extracted from CN-PAGE band II.

Image analysis of 66650 particle projections, selected from electron micrographs of the band II containing photosystem II supercomplexes and megacomplexes, resulted in classification of particles into 64 classes (Fig. S2).

**Supporting information Fig. S3** - Separation of pigment-protein complexes from Scots pine using CN-PAGE.

**Supporting information Fig. S4** – Structural characterization of photosystem II supercomplexes and megacomplexes from Scots pine.

**Supporting information Methods 1** - Proteomic characterization of Norway spruce photosystem II supercomplexes and megacomplexes.

***Protein sample preparation***

Electro-eluted PSII supercomplex and megacomplex samples from the band I and band II (Fig. 1) were mixed with 200 µL of 100 mM triethylammonium bicarbonate buffer pH 8.0 (TEAB) containing 6 M urea, 2 M thiourea and 0.1% (w/v) sodium deoxycholate (DOC) and concentrated by centrifugation at 14,000 g and RT for 15 min in an Amicon® Ultra-0.5 centrifugal filter device (Millipore, Germany) with 3KDa cut-off. The whole procedure was repeated two more times. Then, the concentrates (a total volume of 60 µL) were split into 20 µL aliquots.

***Protein digestion and peptide desalting***

To characterize the protein composition of the investigated complexes, a 20 µL sample aliquot (for both band I and band II concentrated sample) was used for in-solution protein digestion as described previously (León *et al.*, 2013). Briefly, protein complexes were reduced using 5 mM Tris(2-carboxyethyl)phosphine at RT for 30 min, alkylated using 55 mM iodoacetamide at RT in the dark for 30 min, diluted to 200 µL with 20mM TEAB containing 0.1% (w/v) DOC and digested with the use of 1 µg of Sequencing Grade Modified Trypsin (Promega, WI, USA) at 37 °C overnight. Then the digested samples were acidified with formic acid (FA; final concentration of 5% (v/v) and pH of ~2.0) and centrifuged at 20,000 g and RT for 10 min. The supernatants were recovered and the resultant peptides were desalted and fractionated employing a home-made reversed-phase micro column (PTFE capillary, id 200 µm, filled with ReproSil Gold C18, 5 µm particles; Dr. Maisch, Germany) connected to a 25-µL gas-tight micro syringe (Hamilton, NV, USA) according to Franc *et al.*, (2012). First, the whole system was wetted with 25 µL of isopropanol and 80% (v/v) acetonitrile and equilibrated twice with 25 μL of 5% (v/v) FA. The acidified digest was subsequently aspirated into the syringe and loaded on the micro column. Before sample fractionation, the micro syringe was filled consecutively with seven mobile phases consisting of 25 mM ammonium bicarbonate (pH 8) with gradually decreasing acetonitrile content (48-32-24-20-16-12-8% (v/v)). Seven fractions (4 µL each) were subsequently collected, mixed with 21 µL with 5% (v/v) FA and directly analyzed by mass spectrometry (MS).

***Mass spectrometry analysis***

MS analysis was performed employing a tandem mass spectrometer UHR-QTOF maXis (Bruker Daltonik, Germany) connected to a nanoflow capillary liquid chromatography system RSLCnano (Dionex, Thermo Fisher Scientific, CA, USA) via on-line nanoESI source (Bruker Daltonik, Germany). The chromatography system and the mass analyzer were operated with the settings published by Simerský *et al.* (2017).

***Data analysis***

The acquired raw MS data were processed with MaxQuant software version 1.6.10.43 (Tyanova *et al.*, 2016) with an instrument parameter setting called “Bruker QTOF” (Beck *et al.*, 2015) and Andromeda search engine (Cox *et al.*, 2011) and searched against *Picea abies*-specific protein database (Grebe *et al.*, 2019) complemented with 247 sequences of common laboratory contaminant proteins (SI Table 2). All detailed parameters and settings are summarized in parameters text file included in the supplementary data SI_Data_1.zip. Search parameters for Andromeda engine were as follows: enzyme specificity was set to trypsin; 2 missed cleavages were allowed; carbamidomethylation of cysteine was included as a fixed modification and N-terminal protein acetylation, deamidation of asparagine and glutamine and methionine oxidation were selected as variable modifications. The search results were combined and filtered for protein inference and 1% FDR at both peptide and protein level. For the evaluation of abundances of the identified proteins (SI Table 1), the well-established iBAQ method (Schwanhäusser *et al.*, 2011) based on summed peak intensities of all peptides matching to a particular protein was applied. Missing annotations for the identified proteins were assigned by pBLAST homology searches against UniProtKB/Swiss-Prot database with the restriction for *Arabidopsis thaliana* as an organism of choice.

**Figure legends**

Fig. S1. Classification of the dataset of particles from band I (photosystem II supercomplexes).

Result of image analysis and classification of 163447 single-particle projections into 80 classes. Classes were sorted according to the particle number in each class in the descending order.

Number of particles in individual classes was as follows (class number-number of particles): 1-15815; 2-12015; 3-9847; 4-9670; 5-6861; 6-6356; 7-5841; 8-4540; 9-4394; 10-4246; 11-3951; 12-3473; 13-3158; 14-2787; 15-2783; 16-2708; 17-2627; 8-2580; 19-2545; 20-2353; 21-2321; 22-2302; 23-2258; 24-2211; 25-2104; 26-2086; 27-1951; 28-770; 29-1729; 30-1579; 31-1554; 32-1469; 33-1416; 34-1373; 35-1372; 36-1237; 37-1219; 38-1057; 39-1006; 40-982; 41-899; 42-899; 43-875; 44-872; 45-860; 46-844; 47-834; 48-796; 49-786; 50-768; 51-753; 52-718; 53-669; 54-641; 55-640; 56-637; 57-618; 58-606; 59-564; 60-557; 61-545; 62-542; 63-535; 64-496; 65-476; 66-453; 67-434; 68-431; 69-412; 70-354; 71-352; 72-286; 73-285; 74-281; 75-255; 76-248; 77-195; 78-176; 79-170; 80-139.

Fig. S2. Classification of the dataset of particles from band II (photosystem II supercomplexes and megacomplexes).

Result of image analysis and classification of 66650 single-particle projections into 64 classes. Classes were sorted according to the particle number in each class in the descending order.

Number of particles in individual classes was as follows (class number-number of particles): 1-4011; 2-3635; 3-3118; 4-2535; 5-2525; 6-2467; 7-2419; 8-2067; 9-1937; 10-1936; 11-1690; 12-1570; 13-1569; 14-1564; 15-1549; 16-1299; 17-1298; 18-1297; 19-1178; 20-1103; 21-1095; 22-1036; 23-1018; 24-992; 25-965; 26-953; 27-931; 28-916; 29-915; 30-832; 31-782; 32-780; 33-775; 34-724; 35-721; 36-696; 37-667; 38-659; 39-622; 40-615; 41-601; 42-549; 43-547; 44-546; 45-525; 46-522; 47-496; 48-486; 49-453; 50-452; 51-437; 52-434; 53-429; 54-365; 55-344; 56-322; 57-301; 58-276; 59-265; 60-230; 61-218; 62-200; 63-142; 64-49.

Fig. S3. Separation of PSII supercomplexes and megacomplexes from Scots pine using CN-PAGE. Isolated thylakoid membranes were mildly solubilized by n-dodecyl α-D-maltoside. The black and white image represents the chlorophyll fluorescence emission detected from the same gel and enables the identification of PSII supercomplexes due to a higher quantum yield of PSII fluorescence at room temperature compared to the quantum yield of PSI fluorescence. The fluorescence signal was detected through a bandpass filter (690-720 nm); excitation wavelength was 460 nm. The red and blue asterisks indicate high-molecular-weight bands I and II with large PSII supercomplexes and megacomplexes, which were subjected to structural analysis by a single-particle electron microscopy. The bands of lower molecular weight represent different forms of PSII supercomplexes, PSI complex and PSII core complex, and LHCII proteins, respectively.

Fig. S4. Structural characterization of various types of larger PSII supercomplexes and megacomplexes from Scots pine.

(A-F) The largest PSII supercomplexes from Scots pine. The supercomplexes A-C and F were eluted from the band I, whereas supercomplexes D and E originate in the band II (Fig. S3). Projection maps of individual types of the PSII supercomplexes represent the best class averages of (A) 5124, (B) 2182, (C) 12250, (D) 688, (E) 141, 1574 (F) particles.

(G-I) Various PSII megacomplexes from Scots pine. The megacomplexes were eluted from the band II (Fig. S3). Projection maps of individual types of the PSII megacomplexes represent the best class averages of (G) 1876, (H) 442, (I) 1060 particles.
